# The effect of seasonality and weather conditions on human perception of the urban–rural transitional landscape

**DOI:** 10.1038/s41598-023-42014-3

**Published:** 2023-09-12

**Authors:** Marek Półrolniczak, Leszek Kolendowicz

**Affiliations:** Department of Meteorology and Climatology, Adam Mickiewicz University in Poznań, Ul. Krygowskiego 10, 61-701 Poznań, Poland

**Keywords:** Climate sciences, Environmental sciences, Environmental social sciences

## Abstract

Landscape perception research into the impact of seasonally changing landscape characteristics with the simultaneous influence of the weather are rare. Therefore, eye-tracking metrics were calculated (fixation and saccades) for the whole tested landscape, while its areas of interest (AOIs) were established based on clustering methods. Moreover, the gaze pattern was analysed using the Voronoi cells method. To identify significant differences in landscape perception according to various weather and seasonality, nonparametric tests were applied. The significant influence of weather/seasonality and their synergistic influence is noticed. The results indicate a rather complex influence of the types of weather in warmer and cooler seasons. Regardless of the weather type, seasonal changes cause greater visual span and shorter fixations in the warmer season. The fixations and saccades are shorter in the warmer season in two AOI’s during positive weather, but are longer in negative weather during the colder season in most AOI’s. The main reasons for the influence of weather and seasonality on the visual perception include seasonal changes in the landscape, resulting in the appearance of the landscape from more urban to natural and vice versa (phenological changes) as well as lighting changes (due to seasonality and type of weather).

## Introduction

The concept of landscape perception can be defined as a complex psychological process, caused by the landscape view, and analysed from many points of view, e.g. empirical, cognitive, expert or psychophysical^1^. However, it is noteworthy that despite the synergic use of many senses in the perception of one’s surroundings, the most important of these is sight ^2^. According to Gholami et al.^3^ and Guo et al.^4^ the visual perception accounts for about 80% of all types of human perceptions. As noted by Bourassa^5^, visual perception is the ability to interpret one’s surroundings thanks to the light reflected from objects reaching the eyes of the observer, while the interpretation itself is influenced both by the physical characteristics of the environment and the cognitive process between the mind of the observer and surroundings. Moreover, visual perception is shaped by many of the observer’s internal features as well as external factors caused by his current environment. Generally, we can point here to internal factors such as experience, age, worldview, education, human culture, intellectual background and many others, while in the group of external factors simultaneously influencing human senses, the very broadly understood attributes of the landscape^5–9^ including weather conditions may be mentioned^10–12^.

For landscape perception, not only individual/group features, but additionally factors such as daytime and weather should be deemed important for future landscape discussions^13^. Till now we know when it comes to the weather that landscape preferences can be shaped by the weather condition and seasonality^14,15^. The weather influences human senses via synergic external biometeorological stimuli shaping well-being and health^16^. The studies have underlined the synergic influence of manifold stimuli as a biotrophic factor of weather that influences the human vegetative system^10,17^. More importantly, the weather’s influence on the human mind can be related to the visual perception that largely depends on the intensity of sunlight and can therefore modify visual landscape perception^7^. So far two types of weather with a different influence on landscape perception were distinguished: negative weather (connected with the atmospheric low-pressure system), and positive weather (related to the high-pressure system)^11,12^.

The importance of landscape visual quality for physical and psychological recovery, nature planning, and conservation as well as management has been widely recognized^18,19^. The visible landscape affects humans in many ways, including aesthetic appreciation, health, and well-being^20^. Moreover, the same landscape can effect various feelings, and could be perceived differently depending on the observer’s features^21^. This also means that the same landscape can be experienced in varied ways^22,23^. Significant elements of the landscape that influence its perception include shapes that can attract attention (conspicuity), colours, and the orientation of contours^24,25^. These features largely depend on the density of plant communities as well as phenological phases of vegetation that are contingent on the seasons^26^. Huang and Lin^15^ underline that a higher hue landscape variation and the purity or intensity of its colour can cause more fixation. Moreover, they pointed out that a yellow-blue colour field, magenta-green variation, and hue change are important to landscape factors respectively in the mountain, aquatic, and open type areas, while a high hue and chroma variation favour visual fixation. In turn, Buhyoff and Wellman^27^ indicated that the landscape seasonality effect is caused among others by the changing colours and seasonality bias according to interaction between the seasons when landscape was photographed and evaluated. The colours and shapes of the environment (or coupled with seasonality changes) obviously affect the observer in a variety of ways. Taking into account the results of research into the influence of weather^11,12^, as well as seasonality in the landscape perception^15^, these questions may now be asked: do these changes cause significant differences in its perception? The next question follows from the previous: can weather type affect the differences in perception according to season?

Typically, methods based on photography or simulation of landscape representations are used to study, evaluate and manage the visual landscape^28–30^. Additionally, all information about new creation and planned changes in the landscape is usually provided to society and residents using photography^1,23,31^. In each of the aforementioned, it is not possible to fully assess and understand information without caring about the weather’s influence on the observer's perception because the key elements of the weather, especially sunlight, that determine the visual perception of the landscape are more often than not omitted. In our opinion, it is crucial to study the weather’s direct impact on the observer in the natural environment. We emphasise that this is about the synergistic action of all-weather elements (strongly related to seasonality), shaping the psychophysical state of the observer and his visual sensitivity, thanks to which s/he is able to perceive the landscape.

In this study, we focused on the relations of seasonality and weather conditions on the urban–rural transitional landscape perception. That particular type of landscape was chosen because it frequently represents green urban spaces like gardens and parks, the appearance of which is also strongly correlated with seasonal changes in the landscape resulting from changes in the weather. Additionally, we can see the benefits to humans of communing with green areas, such as the previously indicated pro-health effects that improve their physical, emotional, and mental health^32–35^, of which the possibilities are yet to be fully exploited. Furthermore, suburban areas' development has great potential in terms of new urban planning or landscape transformation^36^.

Previous landscape perception research using in situ data collected under the direct influence of the weather approach is rather rare. Nevertheless, research based on a landscape picture investigation inside a building suggests that landscape preferences tend to be influenced by the type of weather and seasonal conditions^14,37^. Moreover, our previous investigations on the basis of an outside experiment indicated a weather influence on gaze pattern that differs, for example, in terms of types of weather or even observer expertise^11,12^.

Taking the aforementioned into account, we can hypothesise that the influence of weather type (positive and negative) could be a valid element in the perception of seasonally changing landscape. The main goal of the research is to address the hypothesis that landscape visual perception is related to seasonality and weather type.

## Research area and methods

### Research area

**Figure 1:**
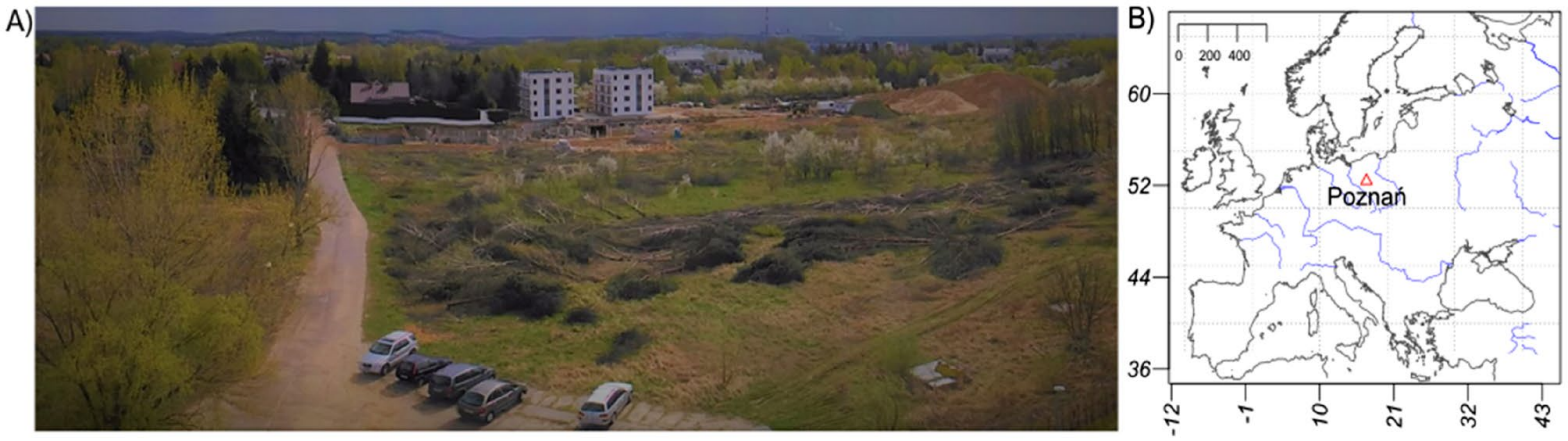
Research scene in spring season (**A**) and location of Poznań on the map of Europe (**B**). The map was created using R programming language ^38^.

The suburban landscape of the northern part of Poznań in Poland (Fig. 1) was selected as a test scenery. Attention should be paid to the location of the research area within a temperate climate zone, which is characterised by a distinct seasonality. Seasonality, in turn, is the cause of changes in the intensity of lighting, weather conditions prevailing in individual seasons, and changes observed in the plant cover. Participants could freely observe the landscape from a viewing point situated on the top of the Collegium Geographicum building. The tested scene is characterised by a scattered, mixed development extended among various types of greenery, therefore it is classified as a suburban or an urban–rural transitional area undergoing urbanisation. For the most part, the investigated panorama's green elements are lawns, shrubs, and trees, i.e. evergreen coniferous and deciduous trees. In turn, the anthropogenic elements of the landscape are mainly scattered low buildings and a construction area in the middle section of the view with two nearly finished blocks of flats. Situated in the foreground is a relatively diversified landscape with a car park, access road, forest, and area with felled trees. In the background, as far as the horizon goes, a landscape of single-family houses and numerous housing estates is dominated by a power station's premises.

### Methods

In our experiment, a group of 24 males and 24 females (48 volunteers) aged between 19 and 25 were investigated. Participants were students of the Geographical and Geological science faculty with regular vision, all of whom were kept in the dark about the goal of the research. Each participant had to complete a personal questionnaire survey concerning age, sex, and current well-being. Persons who were mentally or physically unwell were excluded. All subjects read and signed an informed consent form approved by the Adam Mickiewicz University Ethics Board before the experiment began, the experimental procedure was approved by the institutional review board at Adam Mickiewicz University, and the experiment was performed in accordance with the tenets of the Declaration of Helsinki. Our method of preparing the research group was consistent with those presented in related studies^9,39^. According to the literature, the test group size (48 persons) is sufficient^40^. As pointed out by Eraslan et al.^41^, in eye-tracking research even a group of 20–30 participants may be considered representative.

During the experiment, eye-tracker glasses SMI ETG2 were used. This apparatus allowed for the detection of visual parameters (fixations and saccades) in real-time and dynamic environments. To avoid any measurement errors, participants were asked to remain still without moving abruptly. The position of video frames as well as the gaze map of the corresponding coordinates on a common reference image was determined, which enabled the aggregation of results for further investigations.

The research was conducted in 2017 and 2018 during the warmer (April to September) and colder (October to February) seasons. In total, 8 experiments were carried out, 4 under positive types of weather and 4 under negative ones. With the positive type, we assumed a calm, cloudless weather related to a high-pressure system while negative weather was caused by the action of a low pressure centre and fronts^6,8,11,12^. During the warm part of the year, the favourable weather conditions occurred on May 16, 2017 and June 6, 2018, with unfavourable ones on April 11, 2017 and June 9, 2017. Meanwhile, during the cold season favourable weather conditions occurred on October 16, 2017 and February 26, 2018, with unfavourable ones on November 20, 2017 and January 16, 2018. Before each experiment a 3-dot calibration eye-tracker equipment procedure for each participant was drawn up, after which the main eye-tracking test relay on a free approximately 1 min viewing of the landscape was carried out.

Throughout the viewing of the landscape, the participants’ fixations and eye movements were recorded. The eye-tracking technique allows one to register the eye position, time gaze, as well as speed and direction of the eye movement. The features of looking, called saccades and fixations, have been commonly used as a measure of visual landscape perception^18,23,24,42,43^. According to Poole and Ball^44^, fixations are described as the time of the eyes’ stabilisation when they are acquiring or decoding information. Moreover, during this moment, the eyes have some tremor drifts and microsaccades may occur as well, so usually the eyes are stable and fixating 90% of the time^45^. According to the aforementioned, the minimum length of time describing a moment of eye stationary stabilisation, i.e. fixation, should be determined. Research has shown that open landscapes usually initiate a longer average fixation duration than the enclosed ones^46^. In turn, the mean reading fixation time is estimated at 225 ms (from 50 to 600 ms)^45^. Interestingly, the mean scene perception fixation in line with Rayner^47^ takes 330 ms. According to Jacob and Karn^48^, a typical fixation time is between 100 and 200 ms, and the lower fixation detection time is at least 100 ms^49^. In our research we assume for fixation at least a 100 ms threshold. Next, on the basis of fixation-related metrics like the number of fixations (while excluding the first one which always biases the centre position) and their duration (in ms), the gaze pattern was analysed. The second measure of viewing used in our research was the saccades which complement the fixation^50^. Saccades describe interconnected eye movements (between two consecutive fixations) quickly transferring the eyes to the next position^51^. They are indicating the observer’s search moment for the new information. Since saccades describe for the most part only the extent without any encoding information, additionally we applied the Voronoi method which is useful in terms of landscape object complexity information^52^.

In studies of landscape perception, the use of measures such as fixations and saccades allows for the determination of the basic characteristics of landscape observation (number of fixations, average time, or average distances between fixations). However, these measures also have limitations, as they do not provide information about the range of observation of the examined landscape. For example, fixations clustered in a specific place in the test landscape may yield in calculations the same values as fixations scattered widely, thereby leading to the wrong conclusions. The same is true of saccades, which are ultimately derived from a measure of fixation. Therefore, in this study, it was decided to use an additional measure using Voronoi cell mapping^53^. This method has already been used in research to determine the extent of observation, which has been referred to in the literature as “extent”, “fixation dispersion”, “distribution of gaze intensity” or “spread of search”^54^.

In terms of methodology, mapping Voronoi cells consists of assigning each fixation one cell, which is made up of a cloud of points in space with distances to a given fixation shorter than their distance to another one. It follows therefore that dense fixations will produce small Voronoi cells, while scattered fixations will form large ones. Their areas calculated for fixation in warmer and cooler seasons in positive and negative weather were compared in order to assess the differences using the Mann–Whitney U test. To avoid pseudo-replication in testing the statistical significance of differences, Voronoi cell ranking was utilized. Average ranks calculated separately for each observer were compared.

### Areas of interest

In the present study, the element-based approach concerning the visual perception of manifold landscape parts collected as an Area of Interest (AOI) was used. It could be said that AOI is an area for a specific stimulus/feature and metrics characteristic for that area. Further analysis within these AOIs makes it possible to more easily interpret the eye-tracking metrics^55,56^. Therefore, AOIs have been used in various fields of research: marketing, landscape planning, psychology, etc.^54^. Apart from the AOI’s approach benefits of an easier interpretation, it also has some limitations when used for different types of landscapes. Moreover, in constructing AOIs, the question of how to define their area (size/shape) presents itself^57^. Furthermore, not many AOIs’ instructions are available and these are made differently, hence they are difficult to compare^58^.

In the presented study, AOIs were performed by a clustering of fixations according to the Ward method^59,60^. The grouping was made according to the matrix based on the fixation data. The input dataset of objects (fixation points) were characterised by x/y coordinates. Following the literature concerning fixation^45^, the number of fixation points was limited to those for which the fixation was between 100 and 597 ms (5th and 95th percentile of fixation). Moreover, the landscape observation time was limited to 15 s. Despite eye-tracking experiments, some authors consider it to be a long time of exposure^61^, but in our research the natural environment was examined (weather, landscape). In these circumstances, the observers are under different and more difficult conditions than in the laboratory. Moreover, Manassi and Whitney^62^ showed that human visual stability is related to a 15 s visual past experience.

As a result of the applied Ward’s procedure (99 percentile cutting-off distance of grouped elements^63^, 8 groups of AOIs were created. Next, in order to distinguish the most frequently observed objects in the area of individual AOIs, the method using fixation density distributions in the test landscape along the x and y axes was used. These objects were located at the intersection of the maximum densities for the frequency of fixation distribution in a given AOI (Fig. 4). In the case of a clear bimodal distribution, two points were created, and in the remaining cases one. Furthermore, to assess the differences in landscape perception according to season and weather, the following indexes for all AOIs were used:

- AFD—average fixation duration in ms,
- ASD—average saccade distance in pixels,
- TFD (total fixation duration in percent)—the time spent in the AOI based on the total duration of all respondents’ fixations (excluding data points between fixations) as a percentage of total AOI’s fixation,
- TFN (total fixation number in percent)—the number of fixations in the AOI based on the total number of all respondents’ fixations as a percentage of the total AOI’s fixation number.

## Results

### Visual span

**Figure 2:**
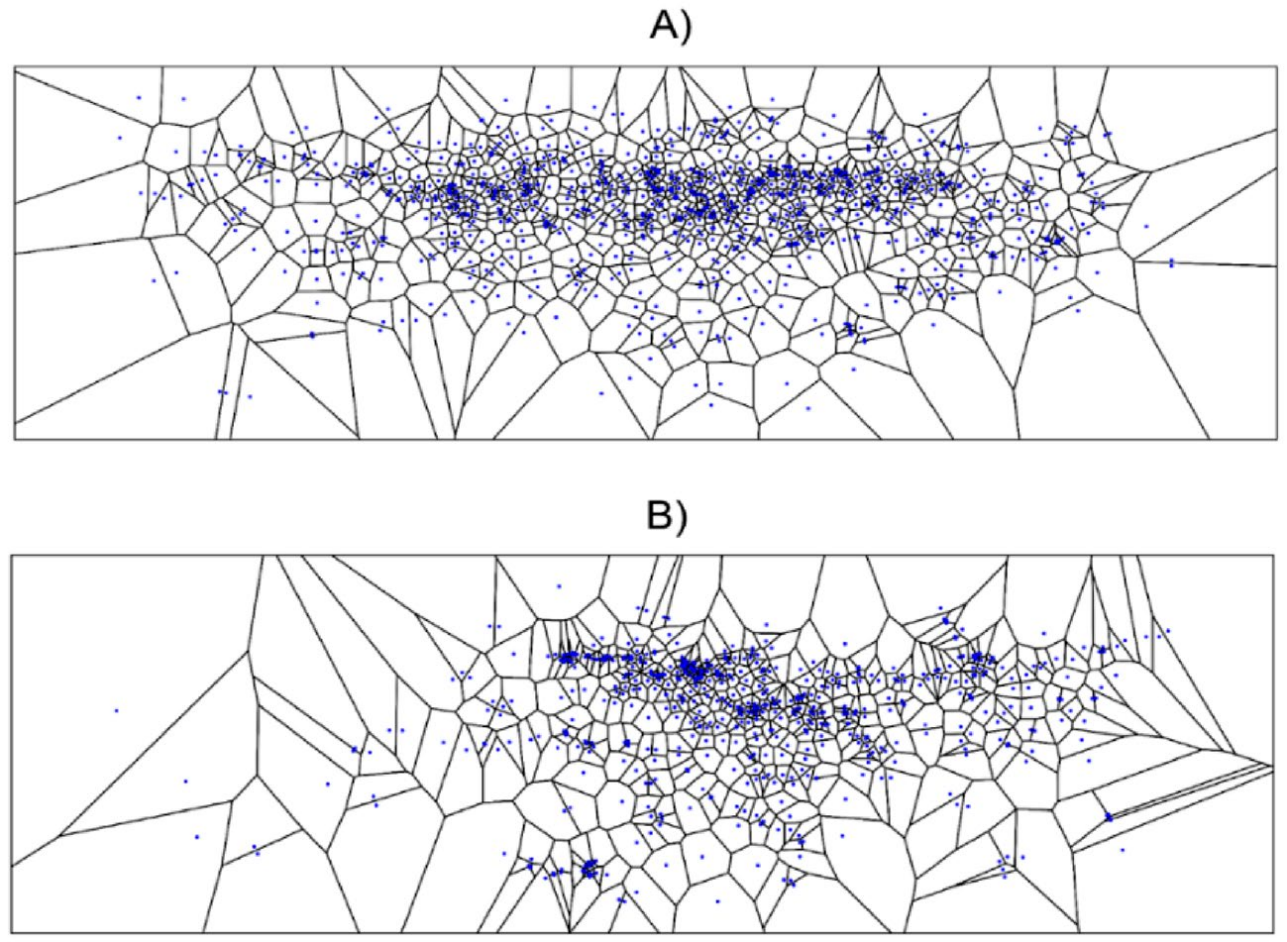
Examples of Voronoi cells in negative (**A**) and positive (**B**) type of the weather in the colder season of the year after the first 15 s of watching.

To examine the visual span during seasons and weather types, the Voronoi cell method was used (Fig. 2). For the warmer season, the Voronoi cells are larger and in a colder season the cells are smaller, which means there a larger gaze dispersion in the warmer season, and a smaller one in the colder season. The detailed results of the Mann–Whitney U tests for all seasons and weather types are shown in Table 1. First, we examined the influence of the season of the year on all participants. The U-test values indicated a significant difference between the seasons (*p* < 0.05). Next, both positive/negative weather types were compared separately for the warmer and colder season of the year. The U-test indicated a significant differences (*p* < 0.05) as well. In summary, the participants of the experiment perceived the test landscape differently in both warmer and colder seasons and in the different weather types in a particular season.

**Table 1:** The Mann–Whitney U-test values (*p*) for the Voronoi cells area in seasons and weather types.

Voronoi cell area	Season		Weather (Warm Season)		Weather (Cold Season)	
	Warm	Cold	Positive	Negative	Positive	Negative
Mean rank	50,31	40,02	25,25	32,92	29.60	37,26
N	31	31	31	31	31	31
P	0,048		0.011		0.031	

Fixation and saccade indexes for the whole scene.

N means the number of observers.

**Figure 3:**
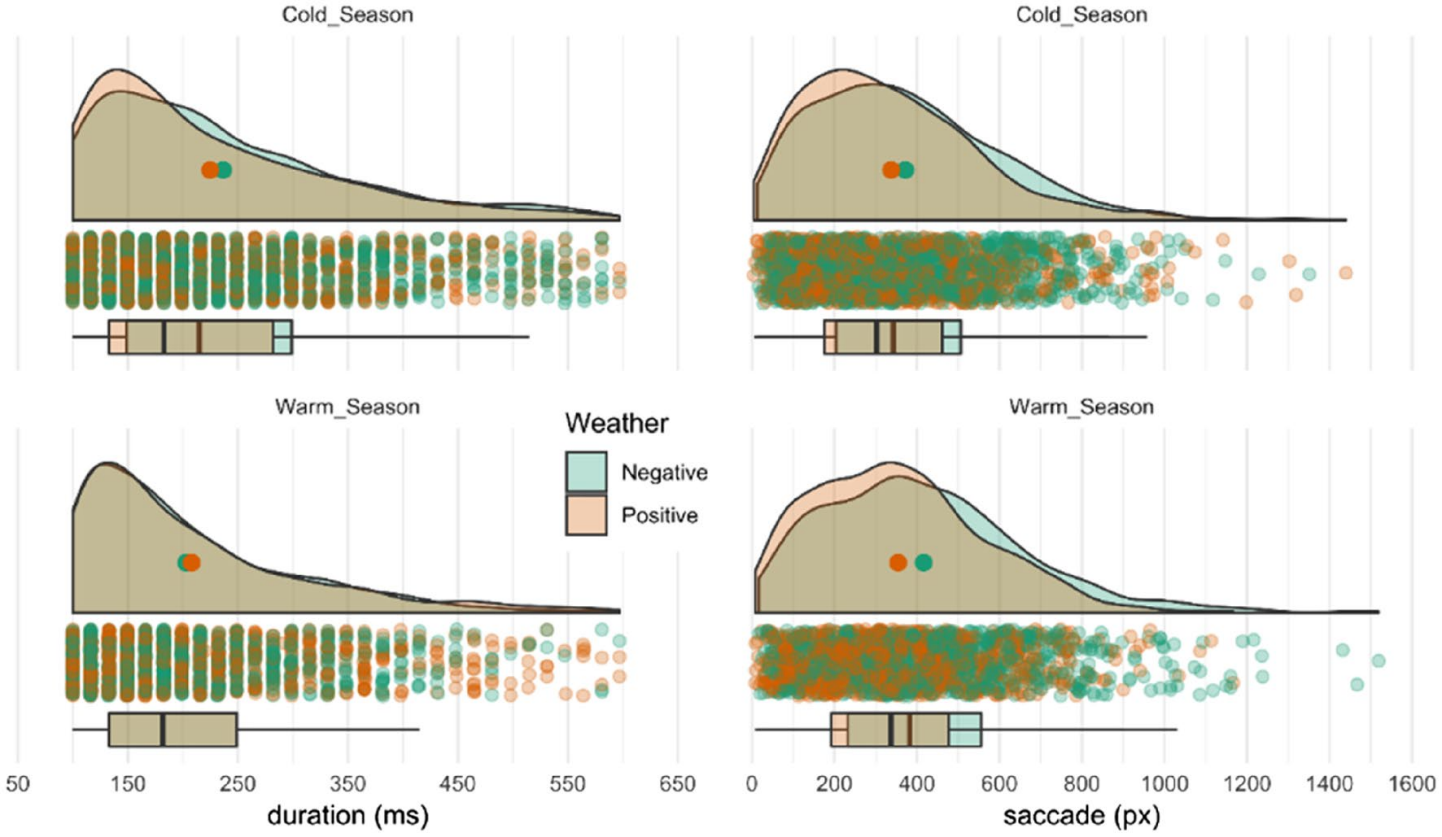
Raincloud plot of AFD (ms) and ASD (pixels) distribution in the cold and warm season in positive and negative weather. The point in the centre of each cloud represents its mean. The rain represents individual data points and boxplots indicate minimum, first quartile, median, third quartile, and maximum value.

The indexes based on fixation and saccade values (AFD, ASD, TFD and TFN) were first calculated for the whole year and then individually for two weather types on the entire landscape (Fig. 3, Table 2—last column). Then, the impact of the seasons (warmer and colder) on landscape perception was examined. The results of this part are presented as probability density distribution in Fig. 3, as well as in Table 3 (last column) as the average values marked in bold or with a box in the case of its significant differences (according to the Mann–Whitney U-test).

**Table 2:** The values of AFD, ASD, TFD and TFN according to a whole year, seasons, and weather types.

	Area of Interest (AOI)								
	1	2	3	4	5	6	7	8	mean/total
Whole year									
AFD	224.8	223.0	212.2	220.5	212.9	254.0	194.3	197.0	217.3
ASD	333.4	346.8	426.3	309.5	348.8	368.0	556.5	470.7	395.0
TFD	25.8	5.1	17.1	21.1	10.8	10.3	4.1	5.7	100.0
TFN	25.2	5.0	17.7	21.0	11.2	8.9	4.6	6.4	100.0
Type of Weather									
Positive									
AFD	**216.8**	**209.6**	207.8	217.3	212.0	249.2	206.5	203.2	**215.3**
ASD	**298.5**	311.6	412.4	**290.3**	**316.6**	341.4	571.2	463.9	**375.7**
TFD	24.6	**3.2**	16.7	21.6	12.6	11.2	4.3	5.8	100.0
TFN	24.6	**3.3**	17.4	21.5	12.8	9.8	4.5	6.1	100.0
Negative									
AFD	**231.6**	**229.0**	215.9	223.5	214.1	259.0	183.9	191.9	**218.6**
ASD	**363.0**	362.5	438.2	**327.4**	**386.9**	396.2	543.9	476.1	**411.8**
TFD	26.8	**6.7**	17.5	20.6	9.3	9.5	3.9	5.7	100.0
TFN	25.8	**6.5**	18.0	20.5	9.7	8.1	4.7	6.7	100.0
Seasons									
Warmer									
AFD	**199.7**	213.2	214.1	**181.5**	208.2	**221.6**	**181.3**	198.3	**202.2**
ASD	335.6	338.6	431.1	298.4	**326.5**	341.8	622.7	477.6	396.5
TFD	**18.2**	8.2	**33.9**	**7.5**	15.0	**4.0**	**1.2**	12.0	100.0
TFN	18.7	7.9	**32.6**	**8.5**	14.8	**3.7**	**1.4**	12.4	100.0
Colder									
AFD	**237.6**	248.6	202.3	**229.4**	220.3	**261.6**	**196.4**	186.8	**222.9**
ASD	332.3	368.0	401.0	312.0	**383.5**	374.1	546.1	418.3	391.9
TFD	**31.5**	2.8	**4.6**	**31.2**	7.6	**15.0**	**6.2**	1.1	100.0
TFN	30.7	2.6	**5.2**	**31.5**	8.0	**13.3**	**7.3**	1.4	100.0

The bold fonts mean significant differences (*p* < 0.05).

**Table 3:**
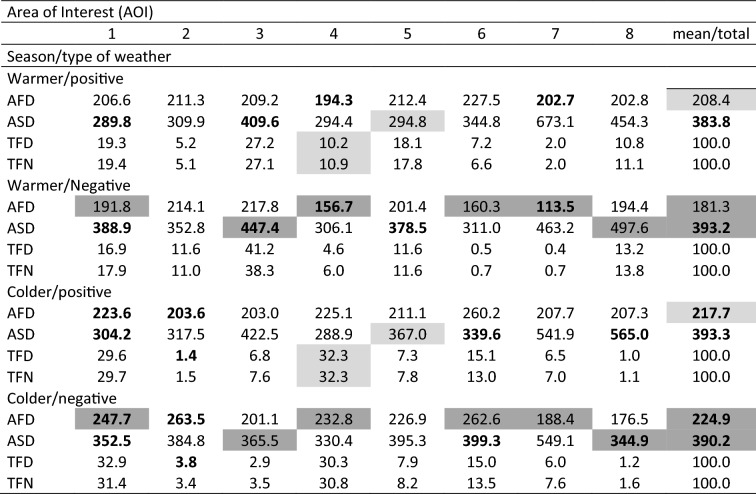
The values of AFD, ASD, TFD and TFN according to season and weather type.

The bold fonts mean significant differences (*p* < 0.05) (inside each of the two panels: warmer season (upper panel) and colder season (bottom panel). The light and dark rectangle mean significant differences (*p* < 0.05) between values of upper and bottom panels. The light rectangles concern differences between warmer and colder seasons in positive weather and dark rectangles concern differences between warmer and colder seasons in negative weather.

The analysis of landscape perception indicators shows that the mean AFD and ASD values for the entire year are respectively 217.3 ms and 395 pixels (Table 2, Fig. 3). The AFD values in positive (215.3 ms) or negative (218.6 ms) weather are respectively lower and higher than the annual mean, while the test statistics indicate their significant differences (*p* < 0.01). Similar relationships, i.e. higher and lower values than the average in the seasons, and statistically significant differences (*p* < 0.001) between these values are observed in the case of ASD (Table 2). Even greater differences from the average for the year occur in both AFD and ASD in seasons. AFD in the warmer season is 202.2 ms and in the colder season 222.9 ms, while ASD is 396.5 and 391.9 pixels, respectively. In the case of both indexes, statistical tests exposed their significant differences in mean values between seasons (*p* < 0.001) (Table 2). In the warmer season, for both negative and positive weather, only significant differences between averages (383.8 and 393.2) were recorded for ASD (Table 3), but in the colder season significant differences were noted in both AFD and ASD. Comparing the seasons (cooler and warmer) while taking into account the types of weather (positive/negative), it can be concluded that there are differences in the case of both indexes. The AFDs in the warmer season are shorter in both positive and negative weather types (208.4 and 181.3 ms accordingly), and are significantly different compared to the corresponding values from the colder season (217.7 and 224.9 ms). On the other hand, the ASD in the warmer season has lower values in the positive weather (383.8 ms) compared to values in the colder season, but has higher values in the negative weather type (393.2 ms) in the warmer season compared to the same type in the colder season (390.2 ms) (Table 3).

#### Areas of interest

**Figure 4:**
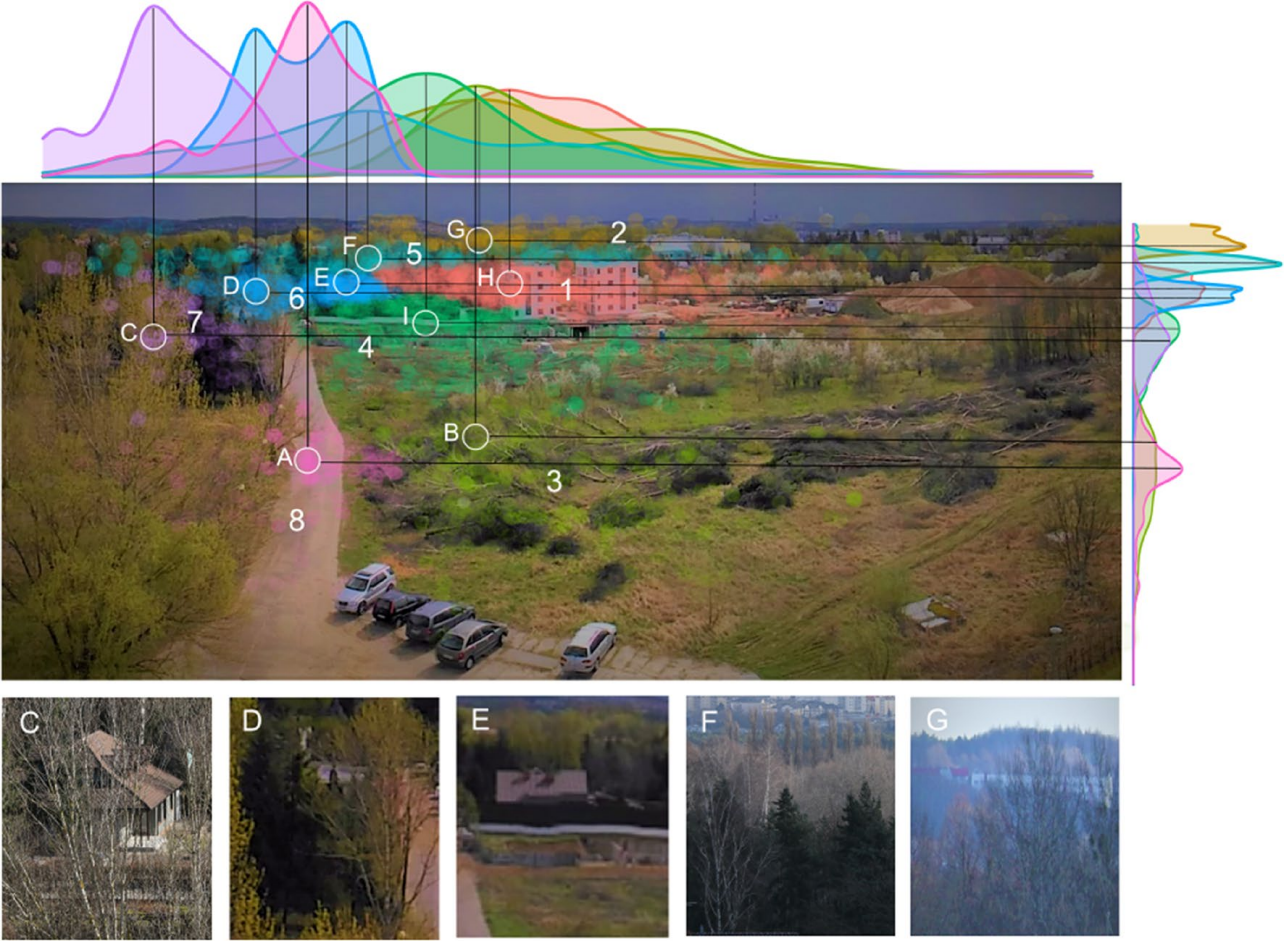
The distinguished AOIs: (1) multi-story buildings, (2) horizon area, (3) tree-felling area, (4) construction area, (5) river valley tree area, (6) single-story buildings, (7) mixed-tree area, (8) dirt road. The areas with the most frequent fixations in particular AOIs are marked in circles according to the density distributions along the x and y axes. C-G pictures zooming in on the indicated areas.

To describe precisely the landscape’s details, the observers paid more attention to, the eight AOIs were established (Fig. 4). AOI 1 at the centre is characterised by the presence of two multi-story buildings located in the background of tall trees. Located in the horizontal part of the landscape, AOI 2 is situated between the Warta River valley and the line of horizon. The elements included in the main features of AOI 2 are a mixture of low vegetation and forest area as well as low or middle building settlements and industrial areas. AOI 3 is situated in the foreground and is created by tree-felling. AOI 4 extends from AOI 3 to AOI 1 and 6. The main features of this part of the landscape is a construction area surrounded by low vegetation. AOI 5 is located under the horizontal AOI 2. It is covered by various kinds of trees growing along the river valley. AOI 6 is surrounded by AOIs 4, 1, 5 and 7. This is an area with conifers and a deciduous mix of trees with scattered low-rise buildings. AOI 7, located on the left side of the scene, is covered by a mixture of different kinds of trees. Finally, AOI 8 (the dirt road and low vegetation area) extends from the foreground to AOI 7 and 4.

First, the use of fixation density distributions along the x and y axes has made it possible to unambiguously determine the most frequently observed elements within each AOI (Fig. 4A–I). In AOIs 1, 2, 5, 6, 7, the most frequently observed objects were buildings and in AOI 4 it was a fence wall extending horizontally from the road to the tallest buildings (Fig. 4). According to the most dense distribution of fixations, only in two AOIs, i.e. 3 and 8, were there no buildings while most often fixations were focused on the middle of the road or on one of many similar objects (cut trees).

On the other hand, in AOI 5 F, the concentration of fixations clearly indicates a place of high contrast associated with a cluster of different tree species. It is worth noting that the seasonal changes in vegetation have allowed the observer in AOI 7 and partly in AOIs 6, 5 and 2 to see buildings covered in the warmer season.

Next, fixation’s and saccade’s statistics were calculated for each AOI created. The calculations were first performed for the whole year and then for the seasons without dividing the weather into different types (Table 2). All calculations were made while taking into account both weather types and seasons (Table 3).

The values of AFD and TFD in individual AOIs are very similar to their annual averages. The AFD range of values for the whole year is from 194.3 ms (AOI 7) to 254.0 ms (AOI 6). In turn, the ASD values range from 309.5 pixels (AOI 4) to 556.0 pixels (AOI 7). The TFD and TFN are mostly similar in particular AOIs, ranging from just above 4.0% (AOI 7) to 25.0% (AOI 1). Furthermore, calculations of all parameters were done separately for positive and negative weather. Taking into account the weather types (positive/negative), the AFD and ASD values in positive weather for most AOIs (1–6) are shorter than in negative weather. Significant differences according to AFD in the perception of individual AOIs concern AOI 2, and for ASD AOIs 1, 4 and 5. The analysis of TFD and TFN indicates their similar participation in individual AOI in both types of weather and for the whole year. Significant differences for these indicators are found only in AOI 2.

**Figure 5:**
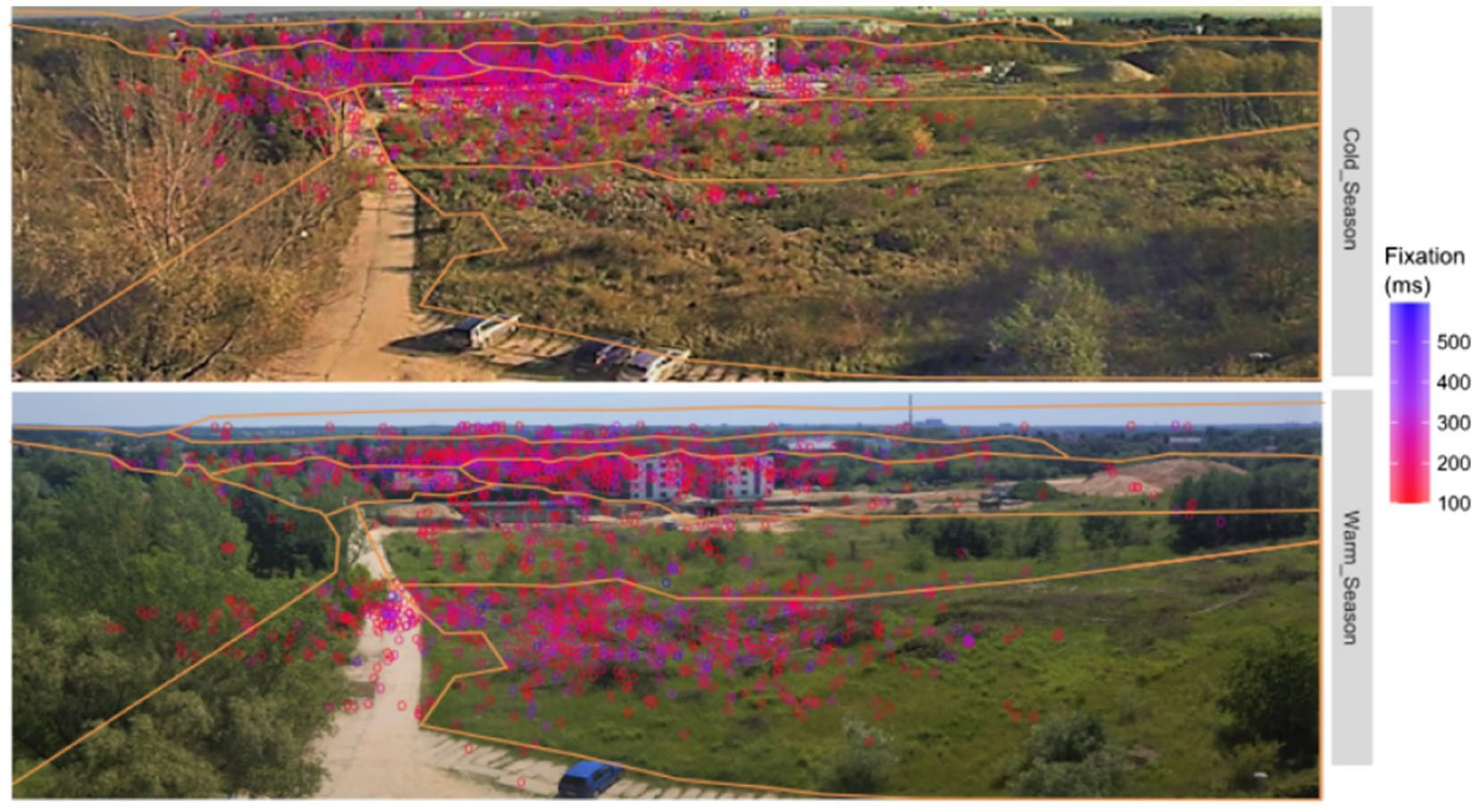
Examples of spatial distribution of fixation on the background of AOI (orange line—approximate range on the base of AOI in Fig. 4) during colder and warmer season in positive weather.

Next, the analysis considered values for the seasons (warmer/colder). Generally, AFD is shorter in the warmer season in the vast majority of AOIs. Only in AOI 8 does the reverse occur. There are significant differences in AOIs 1, 4, 6 and 7 (AFD). According to ASD, in four AOIs (2, 4, 5, 6) the values are lower in the warmer season, while significant differences are found in AOIs 3 and 5. A seasonal change in landscape perception is significantly stressed by the TFD and TFN indices in AOIs 1, 3, 4, 6, 7, as well as 3, 4, 6, 7, respectively. The distinctly different values of TFD and TFN in AOIs 3, 4 and 6 are noteworthy because they indicate sizable differences according to season (Fig. 5).

In further analyses, the type of weather accompanying the observations was also taken into account (Table 3). First, the values of indicators in positive and negative weather during (warmer and cooler) seasons were compared. Then, the indicators were analysed by comparing the obtained values for the same type of weather in different seasons (Table 3). In the warmer season, the AFD values in individual AOIs varied depending on the type of weather. Significant differences occurred in the case of AOI 4. In this case, AFD in positive weather was higher than in negative one—the recorded values were 194.3 ms and 156.7 ms (AOI 4), respectively. In the case of ASD, significant differences were found in AOIs 1and 3. In each case, unlike in AFD, the values in negative weather were higher than in positive weather. The recorded values were respectively 289.98 ms and 388.9 ms (AOI 1) and 409.6 ms and 447.4 ms (AOI 3). In the case of TFD and TFN in the warmer season, no significant differences were found in any AOI due to the type of weather.

Subsequent analyses concerned the colder season (Table 3, last panel). A comparison of values of indicators in positive and negative weather showed that the AFD values were significantly different in AOIs 1 and 2. In both cases, the AFD in positive weather was lower than in negative weather—the recorded values were respectively 223.6 ms and 247.7 ms (AOI 1) as well as 203.6 ms and 263.5 ms (AOI 2). In the case of ASD, significant differences were found in AOIs 1, 6 and 8. In two AOIs (1, 6), the values in negative weather were higher, while in AOI 8 they were lower than in positive weather. The recorded values were respectively 304.2 ms and 352.5 ms (AOI 1), 339.6 ms and 399.3 ms (AOI 6), 565.0 ms and 344.9 ms (AOI 8). In the case of TFD, significant differences occurred only in AOI 2, where the recorded value in positive weather was 1.4%, and in negative weather 3.8%. In the case of the TFN index in the colder season, no significant differences due to weather type in any AOI were found.

The next analyses concerned a comparison of values of indicators used in the same types of weather in different seasons (Table 3, lighter shaded fields). In positive weather, significant differences between the warmer and colder season occurred in AOI 4 and 5. For AOI 4, the TFD and TFN indicators were significantly different. In the warmer season, the values of these indicators were lower. The following values were recorded: 194.3 ms and 225.1 ms, 10.2% and 32.3%, 10.9% and 32.3%, respectively. In AOI 5, only the ASD was significantly different—values in the warmer season (294.8 ms) were shorter than in the colder season (367.0 ms) as well.

A comparison of the index values in negative weather for different seasons showed that AFDs were significantly different in AOIs 1, 4, 6 and 7. As before, the values in the warmer season were lower than in the colder season. These were recorded respectively as 191.8 ms and 247.7 ms (AOI 1), 156.7 ms and 232.8 ms (AOI 4), 160.3 ms and 262.6 ms (AOI 6), 113.5 ms and 188.4 ms (AOI 7). The ASD index was significantly different in AOIs 3 and 8. In both, the values are higher in the warmer season. The following were recorded respectively: 447.4 and 365.5 (AOI 3) as well as 497.6 and 344.9 pixels (AOI 8).

## Interpretation of the results and discussion

In the previous studies the landscape perception have been investigated using mainly pictures or simulations and the influence of the weather on the observer was neglected ^28,29^. In our study we investigated the direct influence of the weather on the observer, which is a considerable methodological and organizational challenge due to difficulties in the organization of research and their synchronization with the selected type of weather (positive/negative). Thus, research planning based on the weather forecast as well as the timing ability of the observers always become a huge challenge. The goal of the study was to explain the impact of seasonality and weather type on the visual perception of an urban–rural transitional landscape. The observed changes in the landscape consisted mainly of changes in phenological appearances (the presence/absence of trees and shrubs foliage and its colours, and the growth of low vegetation in the warmer season) as well as in the lighting of the landscape (changes in the wavelengths of light, its angle and intensity). The aforementioned elements caused changes in the landscape in its elements visible to the observer throughout the year, but also revealed some elements hidden by vegetation during the growth season. For example, the seasonal landscape change revealed or obscured buildings (due to foliage in AOIs 2, 6, 7) or tree felling (growth of grass and shrub partially masking fallen trees in AOI 3). It can be noticed that in these AOIs the gaze is focused precisely on these places. It is difficult to clearly state why this is happening, whether it results from the observer's greater interest in anthropogenic objects or elements unexpectedly seen in the ordered landscape in an urban area (tree felling). It may also have the effect of surprise due to the appearance of 'new' elements in the already known landscape, which can be revealed thanks to seasonal changes. Generally, the concentration of fixations on buildings, tree felling or high contrast trees confirms some regularities already described by two concepts of visual perception. According to the first, i.e. bottom-up strategy^45,64^, visual attention is related to low-level features like rich texture and high colour contrast, as well as complexity^65,66^. The second visual attention concept, i.e. top-down strategy based on semantically meaningful features, is associated among others with education, preference, past experience, and memory^45^. In line with this concept, it can be assumed that frequent fixations in landscape areas obscured by seasonal changes in vegetation may also be caused by a ‘searching’ for remembered objects. Overall, our research confirms the knowledge of viewing strategy as described by the concepts of visual perception. They also supplement it with information on the impact of seasonal changes in the landscape on its characteristic features described in both concepts.

In the presented research, the distribution of fixations revealed differences between warmer and colder seasons. The extent of fixation in the warmer season was significantly greater, which means a larger dispersed gaze pattern in the warmer season, and a more clustered one in the colder season. According to the research of Dupont et al.^9^, the extent of gaze on urban areas is greater than that for semi-urban and semi-rural, which means an increase in the scope of visual exploration along with urbanisation. In our study, we found that the seasonal change in the observed landscape causes more anthropogenic objects to be exposed in the colder season. It can therefore be concluded that the nature of the observed landscape changes into becoming more urban due to seasonality. Accordingly, our research confirms that the extent increases with increasing urbanization, although here it is caused by a change in the appearance of the landscape for phenological reasons. Moreover, Dupont et al.^9^ emphasised that greater dispersion also occurred unexpectedly in rural areas, which does not follow the previously identified trend. In our study, we showed that differences in the fixation dispersion within a given season may depend on the type of weather. Thus, we may conclude that the unexpected wide dispersion of gaze in a rural landscape could also be influenced by the type of weather which the authors did not take into account.

Our examination of the influence of both seasonality and weather on the perception of the landscape revealed more dispersed fixations in the warmer season. During negative weather in the warmer season, the spread of fixations was greater. In our opinion, the level of landscape illumination is decisive for a more clustered visual span in the colder season. Higher contrasts in positive weather favour focusing the attention on the most lit areas (AOI 1—white buildings in the centre of the landscape). In negative weather, the contrasts in the landscape level out, becoming less diverse, thereby rendering all areas more equally accessible in terms of visual exploration. Unlike the colder season, the fixation spread in the warmer season was smaller in positive than in negative weather. We may point out that in the colder season an important role is played by landscape phenological changes reinforced by changes in the intensity and wavelength of light arriving at a smaller angle than in the warmer season. This creates new lighting conditions, emphasising among others autumn colours and contrasts—though this is also due to the long shadows of objects. In positive weather, the structure of the first and second landscape plans (AOIs 3 and 4) becomes more visible, emphasising their elements, e.g. felled trees. The results presented here concerning weather influence on the extent of fixation are only partially consistent with those presented in the previous research, indicating that generally the spread of gaze is shorter in positive weather^12^. Our current research points to a significant effect of seasonal landscape variability on the spread of gaze, which can also be more clustered in positive weather during colder seasons.

Considering the influence of weather on landscape perception, generally noticed were shorter fixation durations and saccades in positive weather for most AOIs. The TFD and TFN indicate for the most part their similar participation in an individual AOI in both types of weather for the whole year. The fixations were significantly shorter in positive weather in the central area with dominant multi-storey buildings and in the horizon area where there are also a lot of buildings. The same can be said in the case of central landscape areas related to multi-storey buildings, but also in a construction area or an area with river valley trees. In trying to explain the above dependencies, it would seem that the cause of improvement in landscape lighting, which is the main factor clearly differentiating the type of positive weather from a negative one, should be indicated. Lighting the landscape enhances its elements, 'leading' the observer's eyes towards them and facilitating the acquirement and conversion of information from the landscape^13^. This is evidenced by shorter fixations, but is also confirmed by shorter saccades, e.g. for areas with a large share of buildings and characterised by a high detail of elements concentrated in a small space. The lighting of the landscape clearly influences the way of looking at the farthest landscape plan, in which the sum of the fixation time and the smaller number of them also confirm the easier perception of the landscape in positive weather. In these results, we see a clear influence of the type of weather on the landscape elements that shape visual perception. On the one hand, according to the categorical approach, we have a significant share of such elements as brightness, heterogeneity and urbanisation^4,46,61,67^; on the other hand, the element of the base approach, i.e. AOI, allowed for detecting significant differences and facilitated interpretation^68,69^.

When considering only seasonal changes in the appearance of the landscape, significantly shorter fixations in the warmer season were noticed in areas with the most urbanised landscape, i.e. multi-story buildings, construction sites and single-story buildings. Moreover, important differences between seasons were found for the TFD and TFN in at least half of the designated AOIs. Probably the reasons should be seen as in the case of a study dealing only with types of weather in better lighting, so as to facilitate the observer’s retrieval of information. The warmer season in temperate latitudes is generally the period of the year with stronger landscape lighting and greater contrasts, especially in the most urbanised of areas. Furthermore, according to our research seasonality changes transform in some way the landscape type into being more or less urban or natural. In line with Shi-Han Huang and Yann-Jou Lin ^65^, people have different preferences/viewing behaviour while gazing at different landscapes. They noticed that magenta-green and hue variation, as well as yellow-blue colour areas are related to fixation count, i.a. in open landscapes. What is more, seasonal changes in the landscape may affect visual perception, such that we perceive it as more or less attractive (natural environment vs urban landscape), therefore they even shape the well-being and regenerative capacity of the body ^70,71^.

The obtained results show that the landscape is observed in a different manner both in the warmer and colder season and in the different weather types. Generally, in the warmer season, the fixations and saccades varied depending on the type of weather. The fixations were unexpected and significantly longer in positive weather in both construction and mixed-tree area. The saccades were then longer in multi-story, fallen tree, and river valley tree area. In these seasons, no differences were found for the TFD and TFN due to the type of weather. In the colder season, the fixations in positive weather were significantly shorter than in negative weather in multi-story building and horizon area. In turn, saccades in negative weather were mostly longer in the central part of the landscape, i.e. multi-story and single story building areas, construction, and dirty road area. In our opinion, longer fixations in the warmer season in the construction area may have resulted from the presence of construction crews and machines, the activity of which could affect landscape observation. In the mixed-tree area, on the other hand, longer fixations could be caused by high contrasts and differentiation in this area. An analysis of the literature shows that longer fixations can mean problems with landscape interpretation or difficulty in getting information ^45,54,72^. On the other hand, shorter fixations in the cooler season in positive weather probably result from better visibility of objects in the area of the horizon and multi-story buildings. According to the literature, shorter fixations in this case may mean easier retrieval and interpretation of information from the landscape, as well as indicating the importance of the perceived objects in these areas. According to Goldberg and Kotval^72^, shorter fixations are usually related to objects that are more interesting, meaningful or important to the observer.

The results concerning a comparison of the types of positive weather in the cooler and warmer season indicate that only in the river valley tree area are there significant differences in fixations and saccades. On the other hand, the results for negative weather types between warmer and cooler seasons indicate significant differences in fixations or saccades almost everywhere except for the river valley. In our opinion, this result confirms the influence of both the weather and season on the perception of landscape, as well as showing the complexity of this issue. As it turns out, we also perceive somewhat significantly the same type of weather in different ways depending on the season. This statement concerns mainly negative weather, therefore we suspect that a significant cause, apart from the most frequently indicated physical weather stimuli, may be additional biometeorological elements related to, for example, the human psyche and mood, which are, after all, largely shaped by the weather. Furthermore, if the physical stimuli of meteorological conditions and visual landscape exploration seem relatively easy to explain—they mostly depend on sunlight intensity^7^—then the impact of weather on the human psyche is more complicated and more difficult to explain^16^ because it depends on many factors related to one’s mental state, well-being and health^6,8,10^. To summarise, probably the explanation for it is the biotropic effect of weather related to its synergic influence on the physical and mental state of the observer.

## Conclusions

According to the eye-tracking experiment relay on observation of the urban–rural transitional area landscape, it can be concluded that as a result of seasonality and weather type the visual landscape is changing along with people's way of looking at it. The obtained results indicate that:

- The visual perception of the urban–rural transitional landscape can be modified by the synergistic effect of seasonality and weather type, as well as by either element independently of each other.
- The visual landscape’s appearance is changing due to the seasonality of phenological elements and lighting conditions which also transform in some way the landscape type to being more or less urban or natural by seasonal cover/exposure of some of its elements (buildings). We observe greater interest in anthropogenic objects, high contrast tree area or elements unexpectedly seen in the ordered suburban landscape like tree felling.
- Regardless of the weather type, seasonal changes cause greater visual span and shorter fixations in the warmer season due to the phenological landscape transformation as well more favourable landscape lighting conditions in this season. Lighting the landscape enhances its elements, 'leading' the observer's eyes towards them, thereby facilitating the acquirement and conversion of information from the landscape.
- Visual perception in the same type of weather differs significantly according to the season. The values of fixations and saccades in positive weather are shorter in the warmer season only in two AOI’s. In turn, the fixations in negative weather are longer in the colder season in most of the AOI’s. A significantly different perception of landscape in the same type of weather depending on the season, especially in negative weather, suggests significant action not only in terms of physical weather stimuli, but also additional biometeorological elements related, for example, to the mental state and mood of observers, which are, after all, shaped by the weather.
- The influence of both seasonality and weather revealed less dispersed fixations in the warmer season in positive weather. It would seem that under these conditions, the level of landscape illumination is the decisive factor in that regard, because the higher contrasts of centrally located elements tend to attract the observer’s focus of attention. In the colder season, a more important role is played by landscape phenological changes reinforced by changes in the intensity and wavelength of light arriving at a greater angle than in the warmer season. This creates new lighting conditions, emphasising, for example, the autumn colours and contrasts along with the long shadows of objects.

The conducted research has shown the important role of both seasonality and weather on the landscape perception. The article focuses on the elements shaping visual perception, i.e. the variability in appearance of the studied landscape, resulting from both seasonality and weather, and the impact of these elements on the mental state and well-being of the human observer. The above elements shape human visual perception, acting on a person separately and simultaneously in a synergistic manner. Due to the multitude of elements and their synergy, the issue of visual perception of landscape still poses considerable challenges for researchers. A proper recognition of this issue could constitute the basis for sustainable local policy-making^73^, and better spatial planning, which in turn would contribute to improving the quality of life due to influence of the surrounding landscape.

## Data Availability

The datasets generated during and/or analysed during the current study are available from the corresponding author on reasonable request.
